# Male-Male Clasping May Be Part of an Alternative Reproductive Tactic in *Xenopus laevis*

**DOI:** 10.1371/journal.pone.0097761

**Published:** 2014-05-21

**Authors:** Heather J. Rhodes, Rachel J. Stevenson, Courtney L. Ego

**Affiliations:** 1 Department of Biology, Denison University, Granville, Ohio, United States of America; 2 Department of Neuroscience, Brown University, Providence, Rhode Island, United States of America; Claremont Colleges, United States of America

## Abstract

Male *Xenopus laevis* frogs have been observed to clasp other males in a sustained, amplectant position, the purpose of which is unknown. We examined three possible hypotheses for this counter-intuitive behavior: 1) clasping males fail to discriminate the sex of the frogs they clasp; 2) male-male clasping is an aggressive or dominant behavior; or 3) that males clasp other males to gain proximity to breeding events and possibly engage in sperm competition. Our data, gathered through a series of behavioral experiments in the laboratory, refute the first two hypotheses. We found that males did not clasp indiscriminately, but showed a sex preference, with most males preferentially clasping a female, but a proportion preferentially clasping another male. Males that clasped another male when there was no female present were *less* likely to “win” reproductive access in a male-male-female triad, indicating that they did not establish dominance through clasping. However, those males did gain proximity to oviposition by continued male-male clasping in the presence of the female. Thus, our findings are consistent with, but cannot confirm, the third hypothesis of male-male clasping as an alternative reproductive tactic.

## Introduction

Reproductive behaviors are shaped by a number of different forces, including physiology, the environmental and social context of mating, as well as intra- and intersexual selection [1]–[3]. Thus, an amazingly diverse range of reproductive strategies and tactics has evolved across the animal kingdom. In many species, individuals of the same sex employ one of a set of alternative reproductive tactics, often in a context-dependent manner, to acquire a mate or achieve successful fertilizations [4]–[6].

Anuran amphibians have long been a model for studying reproductive behavior [7]–[10]. Anurans typically reproduce by external fertilization; a male will hold a female by wrapping his forelimbs around her midsection in a position called amplexus and release sperm as she deposits eggs [11]. Male-male clasping, in which one male clasps another in an amplectant position, has also been observed in a number of anuran species, including the African clawed frog, *Xenopus laevis* [7], [11]–[13]. But what is the function of this behavior? In some species of explosive breeders where there is intense short-term competition for mates, male-male clasping has been observed due to a lack of sex discrimination during mate search [11], [14], [15]. However, male-male clasps are not typically sustained in these instances, as the production of a release call by the claspee can effectively signal and terminate an inappropriate clasp, minimizing the cost of indiscriminate clasping [11], [14], [15]. For example, Marco & Lizana observed that in *Bufo bufo*, male-male clasps never lasted more than 3 seconds; male-female clasps, by comparison, can last hours or days [11], [14].

*X. laevis,* a fully aquatic species native to sub-Saharan Africa, does not fit well into this model of indiscriminate clasping. They are not explosive breeders, but rather have a prolonged breeding season during which females become sexually receptive asynchronously over a period of several months [16], [17], therefore more selective mating behavior would be predicted [7], [9], [11]. Also, male-male clasping can be prolonged in *X. laevis*, lasting minutes to hours, thus the presumptive cost for these clasps would be much higher than the short duration male-male clasps observed in other species. In addition to clear energetic costs and loss of breeding opportunity for the clasper, clasping reduces mobility for both animals involved in a clasp [18]; for this species that would lead to increased difficulty feeding or surfacing to breathe. Finally, in laboratory studies, males exhibit different vocal behavior when they are housed with or clasping another male than when they are housed with or clasping a female, indicating that they can and do recognize the sex of conspecifics [12], [17], [19]. For instance, the clasper in a male-male clasp has been observed to produce a vocalization called a chirp; chirping is rarely observed when a male is clasping a female [19]. Likewise, clasped males often produce a growl, a vocalization females are not capable of making [19]. Therefore, it seems unlikely that indiscriminate clasping will explain male-male clasping in *X. laevis*; however, the model has never been formally tested in this species. Thus, one objective of this study was to test the hypothesis that *X. laevis* clasp different sex conspecifics indiscriminately.

If *X. laevis* are capable of sex recognition, then why clasp another male? The second hypothesis we addressed was that male-male clasping is an expression of competition or aggression, with the clasper asserting dominance over the claspee, thereby increasing his odds of winning future reproductive contests. *X. laevi*s show signs of a male-male social hierarchy in the form of vocal suppression. Males have never been observed to chorus in this species; typically only a single male within a pair or cluster will produce advertisement call at any given moment, and one male will be vocally dominant over time [8], [12], [19]. However a study that systematically examined vocal dominance pairwise in a group of frogs found no correlation between vocal dominance and male-male clasping behavior [12], thus it is not clear if or how male-male clasping behavior might relate to social structure. No prior study has tested how vocal dominance or male-male clasping behavior relates to reproductive success. If male-male clasping is an aggressive or dominant behavior, we would expect the clasper to succeed in mating with a female more often than the claspee.

Our third hypothesis for male-male clasping was that it is part of an alternative reproductive tactic by which the clasper gains proximity to mating events by tagging-along with another male while he (the claspee) mates with a female. Because this species reproduces by external fertilization, the clasper could engage in sperm competition, releasing sperm simultaneously with the claspee, and potentially fertilizing a portion of the eggs. Alternative reproductive tactics including synchronous polyandry and sperm competition by peripheral males have been observed in other anuran species [20]–[25]. This model can also be seen as a variant of a sneaker male, in which a male pursues an alternative tactic to gain fertilizations when faced with high levels of competition [25]. If this is the function of male-male clasping in *X. laevis*, we expect frogs that clasp other males in the absence of a female to be less likely to succeed in clasping and mating with a female in a breeding competition. Instead of pursuing the female directly, we expect to see such a male persist in clasping the other male even when a female is present, or otherwise seek proximity to mating events.

We tested these hypotheses by observing clasping behavior for pairs of males and male-male-female triads in a laboratory setting using a combination of video recording and time-lapse photography. First we determined that clasping dynamics for established male pairings were stable night to night. We then looked for evidence of sex discrimination and sex preference in clasping using the male-male-female triads to test our first hypothesis. We looked for associations between male-male clasping behavior (when no female was present) and reproductive success in a triad to determine if male-male clasping was associated with dominance. And finally, we examined the clasping behavior of males that lost a reproductive contest to look for evidence of alternative reproductive tactics.

## Methods

### Ethics Statement

All animal handling and experiments were conducted in accordance with the Public Health Service Policy on Humane Care and Use of Laboratory Animals and with approval and oversight from the Denison University Institutional Animal Care and Use Committee.

Fifty eight adult male *X. laevis* frogs (ranging in weight from 30.5 to 85.9 g) and 14 adult females (92.8–140.0 g) were purchased from Nasco (Fort Atkinson, Wisconsin) and were housed in large unisex group tanks, 5–10 animals per tank, at 20°C on natural light cycles. Animals were fed Aquamax Carnivorous Fish Diet (Purina) twice per week and tank water was changed the following day using tap water treated with Kordon NovAqua Plus. Experiments were conducted during the summer months (June – August) in Granville, OH.

Each male was paired with another male from a different home tank. Pairs were chosen to be visually distinguishable in low-light conditions; this was primarily done by skin color or density of markings, but if it was difficult to find a suitable pair based on coloration and markings alone, body size or shape was used as an additional distinguishing factor. Behavior was not a basis for pair selection. Each pair of males was placed in a 12 L tank and allowed an acclimation period of at least 24 hours. The behavior of the two frogs was then either video-recorded (Sony Handycam) or photographed once per minute using a webcam (Logitech) controlled by YAWCAM software (yawcam.com) under low-light conditions (a 60-watt lamp behind a Carolina Biological Supply red-650 filter approximately 50 cm from the top of the tank; spectral sensitivity of *X. laevis* falls off rapidly >600 nm [26]).

For all experiments, a behavior was coded as clasping when there was no space between the two frogs’ bodies, with the clasper attaching itself either around claspee’s inguinal region (in traditional amplexus) or around another body part (often a hind limb) of the claspee by wrapping its forelimbs around the body. Videos were analyzed using Observer XT software (Noldus Information Technology) by recording the start and end time of each clasping event, as well as the identity of the clasper and the claspee. Time lapse photographs were analyzed similarly, by recording the behavior shown in each image and calculating the percentage of images that show clasping; thus data are reported as percent of observations (% Obsv.). To allow video and time lapse data to be combined for some analyses, and to allow the techniques to be compared, video data sets were sampled once per minute to create a time lapse data set from the videos, which are also reported as percent of observations (% Obsv.). Median error was <2% when comparing results from time-lapse analyses and video analyses of same videos, owing to the fact that rare, short duration events could be missed with time-lapse. Measures such as clasp duration and clasp count rely on continual observation and thus are only possible with video. Time-lapse photography, however, proved to be a reliable methodology for these relatively inactive animals and provided significant savings of time and money on analyses and analysis software.

To determine if male-male clasping behaviors remained consistent night to night for a given pair, 25 pairs of males were observed for 2 subsequent nights and clasping behavior was quantified. Four pairs of frogs were observed additional nights as well, either 3–4 subsequent nights or three nights spaced over 20 days (males were returned to their separate home tanks between night 4 and night 19). To assure independence in statistical analyses, one frog in the pair was randomly designated as the Test Frog, the other as the Stimulus Frog. Clasping behavior in which the Test Frog was the clasper was quantified from 10∶00 p.m. to 1∶00 a.m.

To examine the relationship between male-male clasping and male-female clasping behaviors, a separate experiment was conducted in which 14 male-male pairs were observed first (Night 1 or N1), then a female was introduced at 10∶00 p.m. on the following night (Night 2 or N2). Clasping behavior for both males in the male-male-female triads was recorded from 10∶00 p.m. to 1∶00 a.m. on Night 1 and from 10∶00 p.m. until 6∶00 a.m. on Night 2 (the longer window allowed us to observe oviposition). For eight such triads, the female was injected with 500 IU human chorionic gonadotropin (Sigma-Aldrich) into the dorsal lymph sac at 5 p.m. to induce sexual receptivity [27] (Receptive Female or “RF”). For all RFs, oviposition began between 2∶00 a.m. and 4∶00 a.m. The remaining six triads utilized females that had no hormonal manipulation and did not oviposit, thus they were assumed to be unreceptive (Unreceptive Female or “URF”). Some males were used for both the experiments to determine consistency of male-male clasping behavior (described in the prior paragraph) and the male-male-female experiment (described in this paragraph), either in the same or different pairings.

Data were analyzed and graphed using Origin Pro 9.0 software (Origin Labs). Non-parametric statistical tests were used due to the non-normal distribution of clasping behavior within the population. Correlation of clasping behavior on different nights was assessed using Spearman’s rank correlation. Sex preference in clasping was assessed with chi-square tests for each of the 28 males, employing Bonferroni correction for multiple comparisons. Comparisons of clasping behavior across different groups of animals or conditions were examined using the Mann-Whitney U test or the Kruskal Wallis one-way analysis of variance.

In accordance with publisher policy, data will be made available upon request.

## Results

While casual observation in lab suggested that different males seemed to engage in male-male clasping behavior to differing degrees, we did not know if male-male clasping behavior was stable over time. Thus, we began by observing clasping behavior of a test male housed with a stimulus male for two or more subsequent nights.

**Figure 1:**
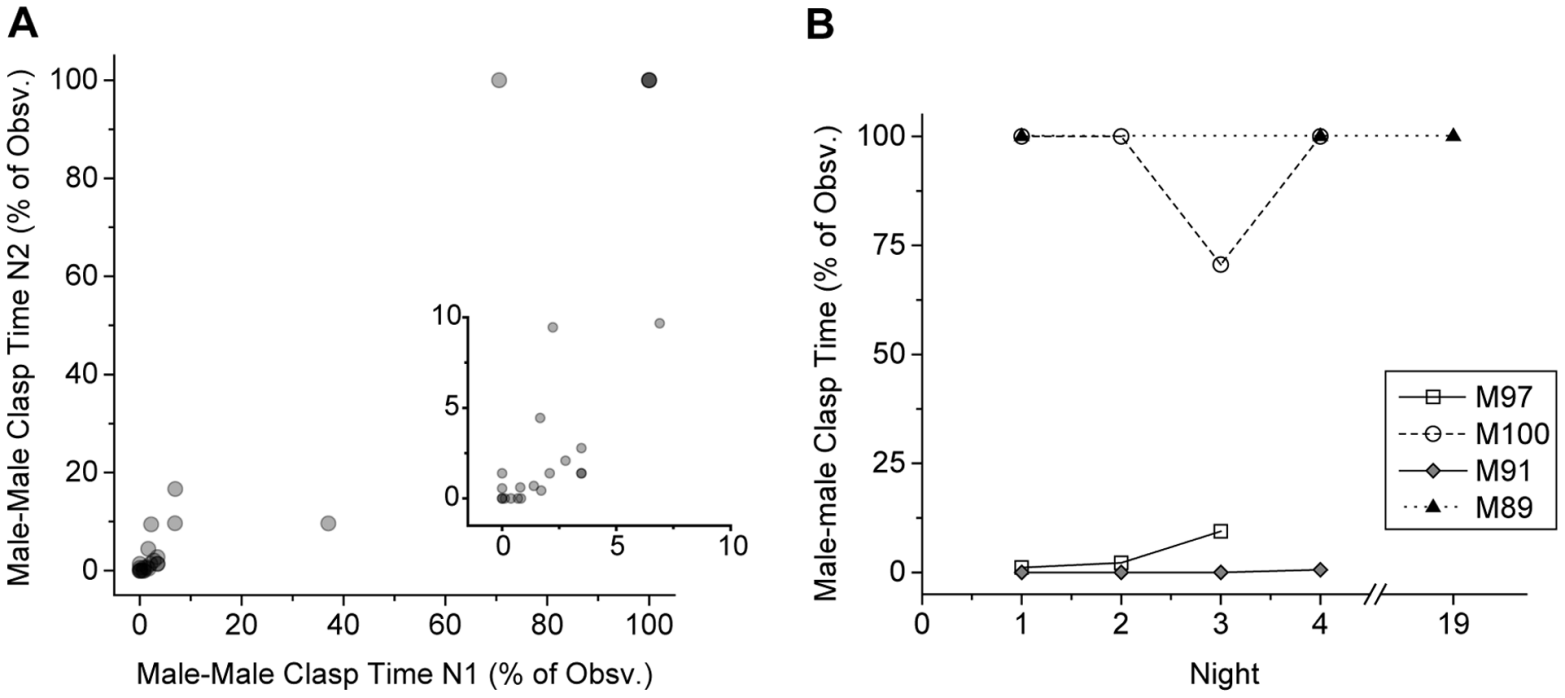
Clasping behavior within male-male pairs is repeated reliably from night to night. **A**. Clasping behavior was observed for one randomly selected male in each pair for two subsequent nights (N1 and N2). Behavior on the two nights was strongly correlated (Spearman correlation coefficient = 0.8783, p<0.001, n = 25). Darker gray symbols indicate multiple, overlapping data points (e.g., there are three data points at 100,100). Inset shows an expansion of the 0–10% range. **B**. Four pairs of frogs were observed on more than two nights, either 3–4 subsequent nights (M97, M100 and M91) or three nights spaced over nearly 3 weeks (males were returned to their separate home tanks between night 4 and night 19). Again, we saw consistent behavior.

We found that 23 out of 25 test males engaged in male-male clasping during the observed time, with 4 animals clasping the stimulus male for the majority of the experiment. Clasping time on Night 1 showed a significant positive correlation with clasping time on Night 2 (Spearman correlation coefficient = 0.8783, p<0.001; Figure 1A). A subset of animals was observed for additional days; all continued to exhibit the same behavioral patterns (Figure 1B). Therefore, clasping dynamics within established male-male pairs seem to be relatively stable night to night.

**Figure 2:**
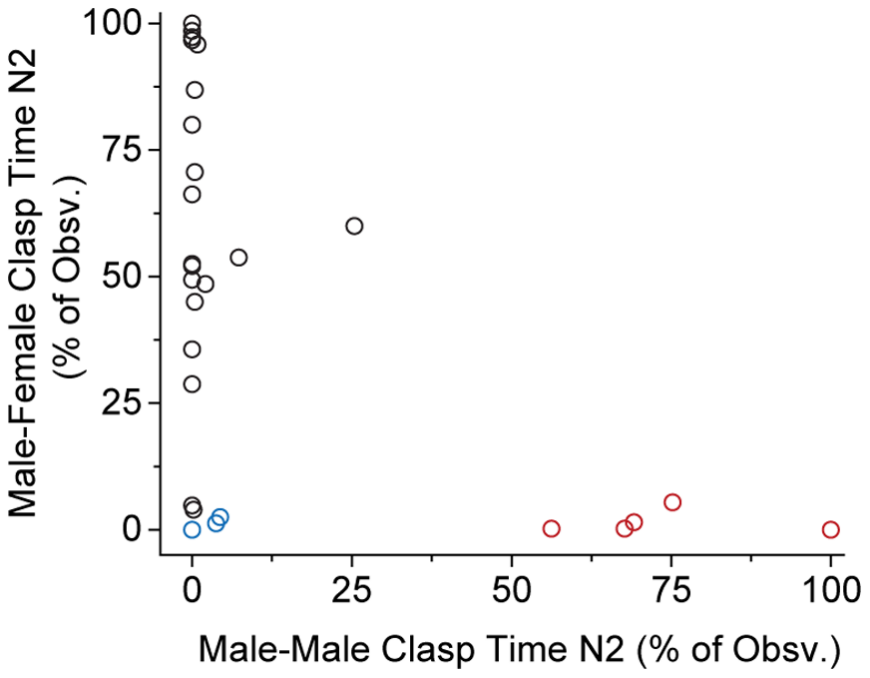
Most male *X. laevis* showed a preference for clasping female conspecifics, but some showed an apparent preference for males and some made little effort to clasp. Examination of the total time spent clasping the male and female conspecifics (measured as % of observations) showed that a subset of males spent considerable time clasping the conspecific male, even in the presence of a female. Red symbols indicate animals that showed a preference for clasping the conspecific male (chi-square tests, p<0.0001); black symbols indicate a preference for clasping the female (chi-square tests, p<0.0001); blue symbols indicate animals that showed no significant preference (p>α).

To test the first hypothesis – that male *X. laevis* clasp indiscriminate of sex – we examined male clasping behavior when males were housed in male-male-female triads. If clasping were indiscriminate we would expect to see males attempt to clasp male and female frogs with equal frequency. We examined clasping preference for each of the 28 males observed using chi-square tests to compare the frequency of male and female clasping across the 480 observations on the male-male-female triad night (to correct for multiple comparisons, Bonferroni corrected α = 0.0018). We found that twenty males showed a clear preference for clasping the female (p<0.0001 for all), five preferred to clasp the other male (p<0.0001 for all), and three did not show a significant preference (p = 0.1149, 0.0122, and 1.0). The three that showed no preference also engaged in little to no clasping of either sex, spending only 6.9, 5, and 0% of observations engaged in any sort of clasping behavior, respectively. These different behavioral patterns are clearly seen in Figure 2. Thus, individual males appeared to show a preference for clasping one sex or the other, inconsistent with the indiscriminate clasping hypothesis.

**Figure 3:**
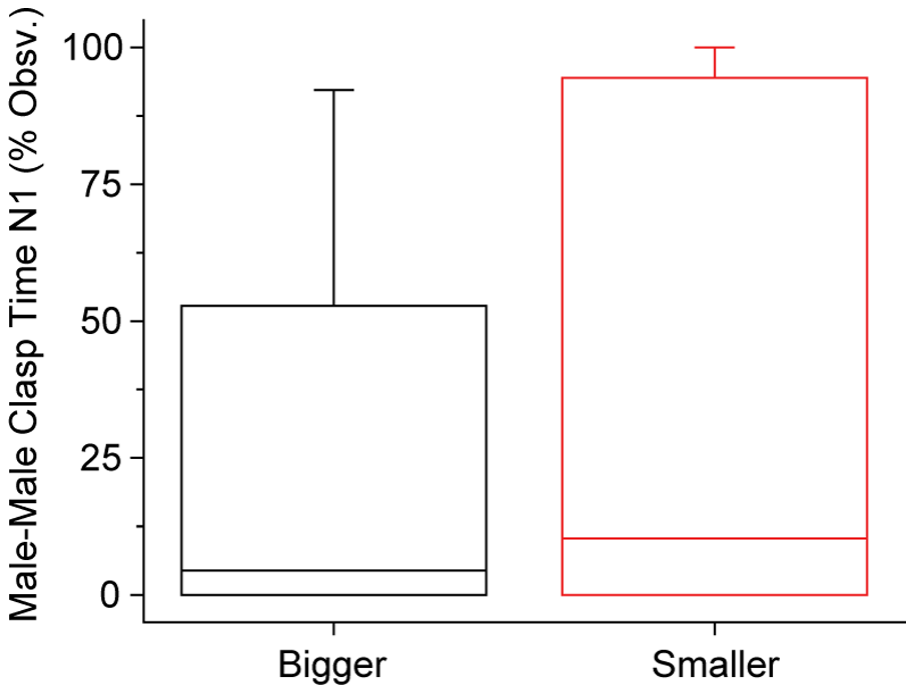
Male-male clasping cannot be explained by size discrimination. The smaller frog in a male-male pair was no more likely to clasp his partner than the bigger frog in the pair (Mann-Whitney U, p = 0.326, n = 14 per category). Box plot shows median, 25^th^ and 75^th^ percentiles; whiskers are 10^th^ and 90^th^ percentiles.

An alternative version of the indiscriminate clasping hypothesis is that males clasp indiscriminate of any direct perception of sex, but instead choose to clasp or not based on the body size of the other frog [15]. Size is sexually dimorphic in *X. laevis*, with females significantly larger than males. Thus, perhaps males simply clasp any frog they encounter that is larger than them because a larger frog has a higher probability of being a female. To evaluate this, we looked at the amount of male-male clasping by relative sizes of the frogs in the male-male pair. Mean weight difference (±SD) within pairs was 7.0±6.5 g (range 0.8–24.3 g). There was no significant difference in clasping between smaller and larger males when size was determined by weight (Mann-Whitney U test, p = 0.326; Figure 3) or snout-vent length (Mann-Whitney U test, p = 0.145; data not shown). Thus male-male clasping does not appear to be strongly driven by size as a proxy for sex discrimination, at least for the range of size differences tested here.

**Figure 4:**
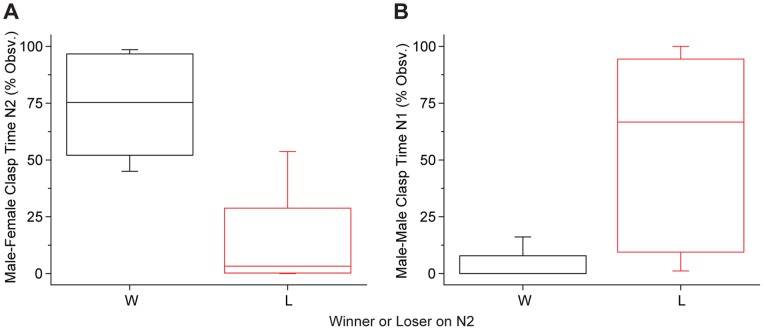
Clasping behavior of “winner” and “loser” males. A) Night 2 clasping behavior was used to determine whether males won or lost the reproductive encounter with the female. The male that predominantly clasped the female in each pair was declared the winner (W); see results for specific criteria. B) Winner males were significantly less likely to have clasped the other male on Night 1 than loser males (Mann-Whitney U test, p<0.001, n = 14 per category) indicating that initiating and sustaining male-male clasps is associated with losing rather than winning primary reproductive access during male-male competition. Box plot shows median, 25^th^ and 75^th^ percentiles; whiskers are 10^th^ and 90^th^ percentiles.

We next investigated the hypothesis that male-male clasping is a dominant or aggressive behavior related to securing reproductive opportunity. We looked to see if male-male clasping on Night 1 was associated with “winning” access to the female on Night 2 when a female was added to the tank. To determine the winner on Night 2, male pairs were assessed for total time spent clasping the female, position of the clasp (around the inguinal region or elsewhere on the female’s body), and amount of time clasping during oviposition (for males housed with RFs only). For most pairs, all measures were in clear agreement with the winning male clasping for more total time (Figure 4A), more time in the inguinal position, and, when applicable, more time during oviposition. In two cases, the paired males clasped the female for similar amounts of time overall; but in both cases, one of the males spent far more time in the inguinal position and was the predominate clasper during oviposition. The amount of male-male clasping on Night 1 was significantly different for Night 2 winners and losers, with winners showing little to no male-male clasping (Mann-Whitney U test, p<0.001; Figure 4B). Thus, contrary to the dominance hypothesis, males who clasp other males are likely *not* to gain reproductive access to the female.

**Figure 5:**
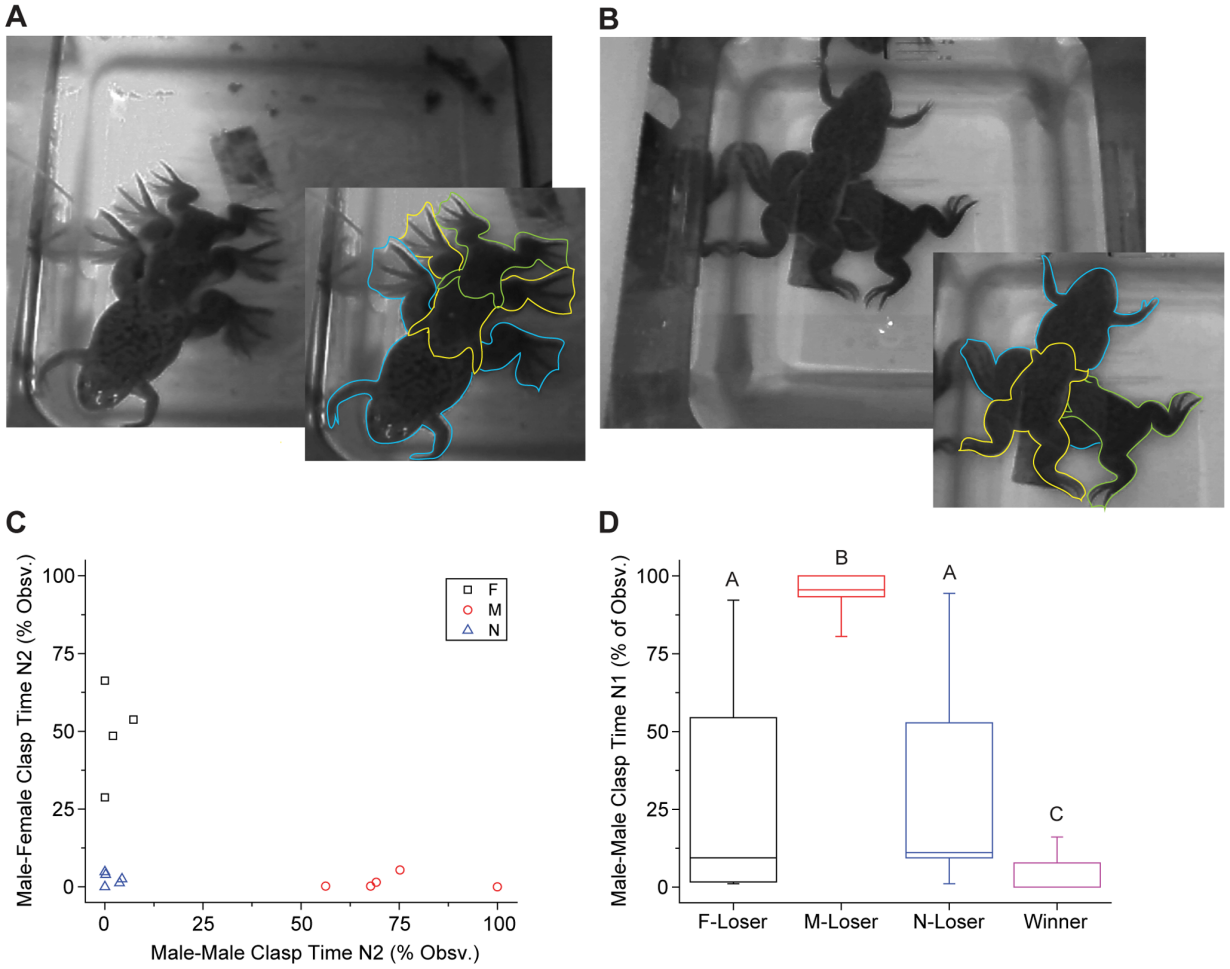
Peripheral males appear to employ different tactics to gain reproductive opportunities. A) The losing male may clasp the winning male or B) he may assume a non-optimal position clasping the female. For both A and B, inset shows outlines of individuals with the female in blue, the winning male in yellow, and the losing male in green. A and B are different triads; eggs from ongoing oviposition can be seen in the upper right corner of A. C) Three distinct patterns of behavior are apparent for losing males on Night 2, Male-directed clasping (M; as seen in A), Female-directed clasping (F; as seen in B) or no/little clasping (N). D) Male-male clasping on Night 1 (with no female present) varied significantly with Night 2 outcome (loser, winner) and with the primary tactic of the loser (Kruskall-Wallis p<0.001, significant differences for post hoc pairwise comparisons indicated with different letters; F: n = 4, M: n = 5, N: n = 5) Box plot shows median, 25^th^ and 75^th^ percentiles; whiskers are 10^th^ and 90^th^ percentiles.

Finally we examined the third hypothesis, that male-male clasping is part of an alternative breeding tactic to gain proximity to oviposition and engage in sperm competition. From the data shown above (Figure 4), it is clear that males which win the reproductive competition rarely if ever direct their clasping behaviors at other males. Males that lose, however, show a range of different clasping behaviors. Qualitatively, we observed that the winning male in the triad often clasped the female in amplexus early in the night and maintained his clasp for most or all of the duration of the experiment. In many cases, the losing male remained close, clasping the amplectant male or the female’s hind limb (Figure 5A, B). Although in some cases, the second male made little physical contact with either the male or female.

To quantify this, we examined how losers spent their time on Night 2 and defined three distinct tactics: those that directed clasps primarily toward the female (F), primarily toward the other male (M), or those that did little to no clasping of either (N) (Figure 5C). Notice that there were no males that devoted time equally to clasping both the male and the female. Thus males that chose to clasp seemed to pursue distinct tactics and/or show distinct preferences for clasping the female or the male, either of which could bring the male into proximity with oviposition.

Different tactics on Night 2 were associated with different behavior in the male-male condition on Night 1 (Figure 5D). Specifically, the male-directed tactic on Night 2 was preceded by a high degree of male-male clasping on Night 1, differing from all other groups (Kruskall-Wallis, p<0.001, followed by post hoc pairwise comparisons, p<0.05). Losing males that engaged in the female-directed tactic or did little to no clasping on Night 2 showed highly variable behavior on Night 1, but both groups showed significantly more male-male clasping on Night 1 than the winners (Kruskall-Wallis, as above, followed by post hoc pairwise comparisons, p<0.05). Thus, while we cannot confirm that male-male clasping represents a reproductive tactic in this species, the current data are consistent with this third hypothesis.

For the male-male-female triads described above, six included unreceptive females and eight included receptive females. No difference was seen in the total amount of time that males clasped females around the inguinal region between URFs and RFs across the eight hours of observation (median, 25^th^–75^th^ percentiles for URFs: 89.2 min, 4.5–234.4 min; for RFs: 231.4 min, 101.8–361.4 min; n = 20 males with video data, Mann-Whitney U test p = 0.272). But for URFs, clasps were significantly shorter in duration (median, 25^th^–75^th^ percentiles for URFs: 1.0 min, 0.6–2.7 min; for RFs: 46.5 min, 24.3–76.1 min; n = 20, Mann-Whitney U test p = 0.003) and greater in number (median, 25^th^–75^th^ percentiles for URFs: 9, 4–41; for RFs: 4, 2–5; n = 20, Mann-Whitney U test p = 0.017). URFs likely induced release with the production of a release call and/or body movement (female leg extensions and barrel rolls were observed but not quantified). Males, however, were persistent in reestablishing clasping. Despite the turnover in clasping, one male was always a clear winner in URF cases, and losing males adopted all tactics (M, F, and N) with similar frequency in RF and URF cases.

## Discussion

In this series of laboratory experiments, male *X. laevis* did not engage in indiscriminant clasping, nor did they assert dominance or increase the probability of mating by clasping a conspecific male. Rather, the majority of males engage in little to no male-male clasping while a subset of males engaged in prolonged male-male clasps with or without a female present. The latter subset was far less likely to successfully achieve amplexus with the female, but most did stay in proximity to the mating event by clasping the amplectant male. From that position it is possible that peripheral males could engage in sperm competition, a hypothesis which will require further investigation.

We showed that males do seem to discriminate between sexes when clasping, with most frogs preferring to clasp females, but a proportion preferentially clasping other males. Frogs are likely using auditory, and possibly chemical cues, to make this discrimination [28]–[31]. Visual cues are unlikely given that these frogs live in turbid ponds, reproduce at night, and their eyes seem to be adapted for detecting looming predators rather than conspecifics [16], [28], [32].

We also showed that male-male clasping is not an aggressive or dominant action; rather, males that engage in little or no male-directed clasping with no female present are more likely to successfully win primary reproductive access to the female on a subsequent night, while males that engage in male-directed clasping lose consistently. Numerous species of anurans engage in physical confrontation with competing males that take a variety of forms, including wrestling, clawing, and pressing the opponent to the ground [11]. But male-male clasping differs from these other physical competitions. While being clasped may impair mobility of the clasped male [18], it does not eliminate his ability to clasp a female. It is the clasper that gives up his ability to engage a female in amplexus.

Thus, we suggest that male-male clasping may be a reproductive tactic to maintain proximity to reproductive events and engage in sperm competition. Evidence of sperm competition and synchronous polyandry is accruing in a diverse set of anurans, including numerous species in at least seven families thus far [24]. In many of these cases peripheral males join amplexus with a single female, similar to what we observed in *X. laevis* [24], [25]. These tactics generally appear to be context-dependent, allowing short-term, opportunistic behavioral choices [6], [25], [33]. Opportunistic changes in calling behavior have previously been observed in *X. laevis* in response to playback of female calling, indicating that these frogs do have flexible reproductive tactics [29], [34]. Anecdotally, one of the *X. laevis* males in the present study that showed a strong preference for male-male clasping, even when an RF was present, was placed alone with an RF for an unrelated experiment shortly thereafter; he clasped the female promptly and sustained the clasp for hours when no other male was present, supporting the notion that these tactics are plastic and context dependent.

Given that sustained male-male clasping does not occur with every pair of males, differential characteristics of the particular males must determine the clasping relationship. These could be characteristics of the clasper (hormonal state, ability to attract a female, or reproductive history, for instance) or of the claspee (hormonal state, ability to attract a female, or tolerance of being clasped). Male-male clasping may in fact be a subordinate behavior that occurs in some male-male pairings. We did not test males in multiple different pairings (as in [12]) and thus cannot assess how stable male behavior is when pairings are changed or if there is a social hierarchy.

If clasping males are indeed engaging in sperm competition, why might clasped males tolerate this interference by another male? We cannot answer this question at this time, but we can offer some possibilities. First, the clasped male may not be able to disrupt the clasp or the cost of disrupting it may be too high; for instance attempting to force release by the clasping male may have a high energetic cost or may risk disrupting amplexus with a female. Alternatively, there may be a benefit to the clasped male through cooperative breeding [5], [35]. Clasping males have been observed to produce advertisement calls [19] thus they may contribute to advertising, relieving some of the cost of advertisement from the clasped male. Likewise if *X. laevis* produce pheromones, as some other anurans have been shown to do [31], the combined chemical signal from two males may increase the likelihood of attracting a female, providing both males with increased odds of reproductive success. The clasped male could also benefit from reduced competition. If one male clasps another, he may essentially be signaling that he is taking the peripheral male position, giving the clasped male better odds of achieving the ideal amplectant position with less competition, presumably for the price of a portion of the fertilizations.

To truly understand male-male clasping behavior we will need a richer understanding of *X. laevis* social structure. There is evidence of complex social interactions and vocal dominance between males both in *X. laevis* and *X. borealis* [8], [12], [19], [36], but their social structure and social hierarchies are poorly understood. For instance, there is no direct evidence as to whether or not *X. laevis* are territorial, and the role of female choice remains unclear [12], [16]. This paucity of understanding is largely due to the difficulty of observing behavior in their natural environment. They are typically found living in the mud at the bottom of turbid ponds and are most active at night, making visual observation exceedingly difficult [16], [32]. Monitoring behavior through audio recording also has limitations in the field; calls do not vary measurably between individuals, and there are no outward motions or other visual cues associated with call production [19], making it impossible to track which individual is calling in a large group using typical audio and video equipment.

Further studies will be needed to confirm or reject the hypothesis that male-male clasping is part of an alternative reproductive tactic. These could include monitoring sperm release of clasping and peripheral males in triads [21], or establishing paternity of offspring resulting from male-male-female triads [23] and relating that to clasping behavior and position of the males during oviposition. Examining additional elements of behavior, such as vocalizations, and behavior in larger, more naturalistic settings would also be valuable.

In conclusion, we found evidence that *X. laevis* do show sex discrimination in clasping with most males preferentially clasping a female when placed in a male-male-female triad, but some males preferring to clasp the other male; thus we reject the hypothesis that male-male clasping in this species can be explained as a product of indiscriminate clasping. We also found that engaging in male-male clasping in the absence of a female was associated with losing a subsequent reproductive contest, allowing us to reject the hypothesis that males that clasp other males exhibit dominance in a male social hierarchy. Instead we found that males that engaged in male-male clasping often continue to do so when a female is introduced, or otherwise maintain physical contact with the amplexed pair, possibly to engage in sperm competition as an alternative reproductive tactic.
